# Development of Polydimethylsiloxane Substrates with Tunable Elastic Modulus to Study Cell Mechanobiology in Muscle and Nerve

**DOI:** 10.1371/journal.pone.0051499

**Published:** 2012-12-11

**Authors:** Rachelle N. Palchesko, Ling Zhang, Yan Sun, Adam W. Feinberg

**Affiliations:** 1 Department of Biomedical Engineering, Carnegie Mellon University, Pittsburgh, Pennsylvania, United States of America; 2 Department of Materials Science and Engineering, Carnegie Mellon University, Pittsburgh, Pennsylvania, United States of America; 3 Department of Ophthalmology, Louis J. Fox Center for Vision Restoration, University of Pittsburgh School of Medicine, Pittsburgh, Pennsylvania, United States of America; 4 Key Laboratory for Biomechanics and Mechanobiology of Ministry of Education, School of Biological Science and Medical Engineering, Beihang University, Beijing, China; University of Minho, Portugal

## Abstract

Mechanics is an important component in the regulation of cell shape, proliferation, migration and differentiation during normal homeostasis and disease states. Biomaterials that match the elastic modulus of soft tissues have been effective for studying this cell mechanobiology, but improvements are needed in order to investigate a wider range of physicochemical properties in a controlled manner. We hypothesized that polydimethylsiloxane (PDMS) blends could be used as the basis of a tunable system where the elastic modulus could be adjusted to match most types of soft tissue. To test this we formulated blends of two commercially available PDMS types, Sylgard 527 and Sylgard 184, which enabled us to fabricate substrates with an elastic modulus anywhere from 5 kPa up to 1.72 MPa. This is a three order-of-magnitude range of tunability, exceeding what is possible with other hydrogel and PDMS systems. Uniquely, the elastic modulus can be controlled independently of other materials properties including surface roughness, surface energy and the ability to functionalize the surface by protein adsorption and microcontact printing. For biological validation, PC12 (neuronal inducible-pheochromocytoma cell line) and C2C12 (muscle cell line) were used to demonstrate that these PDMS formulations support cell attachment and growth and that these substrates can be used to probe the mechanosensitivity of various cellular processes including neurite extension and muscle differentiation.

## Introduction

Over the past decade it has become evident that the mechanical environment has a profound effect on cell survival, proliferation, adhesion, differentiation and metabolism [1]–[8]. Pelham and Wang reported in 1997 that focal adhesion formation and migration of cultured rat kidney epithelial cells and 3T3 fibroblasts were regulated by the stiffness of polyacrylamide (PA) gels [2]. In 2006 Engler et al demonstrated that mesenchymal stem cell specification on collagen coated PA gels was directed towards neurons, muscle and bone on substrates that matched the elastic modulus of these tissues [1]. The insights gained from these types of studies have been extended into other areas where the mechanical environment is now recognized as an important factor. Recent work in cancer biology has revealed that the extracellular matrix (ECM) in tumors is characterized by increased stiffness and that ECM remodeling can lead to invasion and metastasis [9], [10]. Stem cells are similarly sensitive to ECM and substrate mechanics [8], where control of stiffness can drive differentiation into specific lineages [1], [7] or maintain stem cells in a pluripotent state [6]. The commonalities between these studies are experimental tools that control the mechanical environment of cells by modulating the stresses and/or strains cells sense and respond to. Understanding this underlying mechanobiology is important in order to develop improved platforms for *in vitro* cell analysis, tissue engineering scaffolds and regenerative medicine strategies.

As the importance of the mechanical environment on cell behavior has been realized, researchers have developed a number of materials systems to probe these interactions. PA gels have been widely used to create substrates with elastic moduli (*E*) in the range of ∼0.1 kPa to ∼100 kPa, covering the range of many types of soft tissues in the body [1], [2], [6], [11]–[15]. Other types of hydrogels have also been used over a similar stiffness range including synthetic systems based on polyethylene glycol [16] as well as naturally-derived polymers including hyaluron [17], [18], methylcellulose [19], dextran [20], gelatin [21] and fibrin [22]. However, many tissue structures in the body are stiffer than these materials including dense ECM structures such as many types of basement membranes (*E* ∼1 MPa) [23], [24]. Further, at the tissue-scale cells may experience an effectively stiffer environment, such as arterial walls (*E* ∼800 kPa) and cardiac muscle under physiologic blood pressures (left ventricle at peak systole, *E* ≈ 30–400 kPa) [25], [26]. Rubber-like elastomers have elastic moduli in this range including polydimethylsiloxane (PDMS) [27]–[29], poly(n-butyl) acrylate [30] and polyesters [31]. However, the chemistry, water content and surface energy of these elastomers is substantially different then the softer hydrogels and have different synthesis and processing requirements. To date, no single material has been able to effectively cover the entire range of soft tissue elastic moduli from approximately 1 kPa to >1 MPa without changes in other major surface and/or bulk properties known to influence cell behavior. Thus, to fully understand the role of mechanics on cell function it would be ideal to tailor the elastic modulus without altering other material properties such as surface energy, chemistry and roughness.

Here we report development of PDMS substrates where the elastic modulus can be easily and independently tuned to mimic soft tissues over a three order-of-magnitude range. To do this we have blended together two commercially available PDMS elastomers, Syglard 527 and Sylgard 184, in various mass to mass ratios to create low stiffness gels that transition to higher stiffness elastomers. Sylgard 527 and Sylgard 184 are each prepared to manufacturer’s specifications, preserving the stoichiometry of the crosslinking process during gel/elastomer formation. This is in distinct contrast to the reduction of crosslinker commonly used to decrease the elastic modulus of Sylgard 184, which leaves a large proportion of free polymer in the system that can leach out. We demonstrate that the elastic modulus can be controlled without altering surface roughness, wettability, and protein adsorption, material properties that can influence cell behavior. Further, we establish that multiple cell types can be cultured on these surfaces using the PC12 cell line as a model of neural cell adhesion and growth and the C2C12 cell line as a model of skeletal muscle cell adhesion, growth and differentiation. These examples will illustrate the complex role substrate elastic modulus in combination with surface chemistry and micropatterning has on cell behavior.

## Methods

### Fabrication of Polydimethylsiloxane Substrates with Tunable Mechanical Properties

Commercially available PDMS, Sylgard 527 gel and Sylgard 184 elastomer (Dow Corning), were blended to create PDMS substrates with tunable mechanical properties. Sylgard 527 was prepared per manufacturer’s directions by mixing equal weights of part A and part B in a Thinky-Conditioning mixer (Phoenix Equipment Inc, Rochester, NY, USA) for 2 minutes at 2000 RPM followed by 2 minutes of defoaming at 2000 RPM. Sylgard 184 was prepared per manufacturer’s directions by mixing 10 parts base to 1 part curing agent using the same mixing and defoaming cycle. Four different mass ratios of the Sylgard 184∶527 were evaluated; 5∶1, 1∶1, 1∶5, and 1∶10. Each blend was mixed by first preparing pure Sylgard 527 and 184 as described above, and then combining by the indicated mass ratio followed by an additional mixing and defoaming cycle. Once mixed, the PDMS was either poured into 150 mm diameter petri dishes to create ∼2 mm thick films for mechanical testing or spincoated onto 25 mm diameter glass coverslips at 4,000 RPM to create ∼15 µm thick films. All PDMS was cured at 65°C overnight (12–24 hours) for all experiments. Previous studies have reported that this cure time and temperature are sufficient to cure the PDMS such that mechanical properties are constant throughout our experimental protocol [32]. PDMS coated coverslips were treated in a UV-Ozone cleaner (Novascan Technologies, Ames, IA, USA) for 15 minutes before protein coating or microcontact printing.

### Microcontact Printing of Extracellular Matrix Proteins

Lines of fibronectin (FN) or laminin (LAM) were microcontact printed onto the PDMS substrates using an adaptation of previously reported techniques [33]. Briefly, 20 µm wide, 20 µm spaced lines were designed using AutoCAD software and printed onto a transparency-based photomask. Glass wafers were spincoated with SPR 220.3 positive photoresist (Microchem, Newton MA), exposed to UV light through the transparency-based photomask, developed using MF-319 developer (Microchem) and post baked at 115°C for 90 seconds. PDMS stamps for microcontact printing were prepared by mixing Sylgard 184 per manufacturer’s directions (as described above), pouring the prepolymer over the patterned glass wafers and curing overnight at 65°C. Once cured, the PDMS was peeled from the wafer, cut into 1 cm^2^ stamps and examined under phase contrast microscopy to ensure successful pattern development. The PDMS stamps were sonicated in 50% ethanol for 30 minutes and dried using a nitrogen gun and then coated with 200 µL of 50 µg/mL LAM or FN (BD Biosciences, San Jose, CA) dissolved in sterile deionized water. The FN consisted of 60% unlabeled protein and 40% protein labeled with Alexa Fluor 546 Maleimide using an adaptation of previously published techniques [34]. The PDMS stamps were incubated with either LAM or FN at room temperature for 1 hour to allow for the protein to coat the stamps. The PDMS stamps were then rinsed in sterile deionized water (ddH_2_O) and dried using a nitrogen gun before being placed patterned side down on the PDMS coated coverslips. After approximately 5 minutes the PDMS stamps were removed leaving behind the patterned protein. PDMS substrates micropatterned with fluorescent FN were used to validate proper protein pattern transfer across the different blends and were imaged using a Zeiss LSM 700 confocal microscope (Carl Zeiss, Inc., Thornwood, NY, USA). PDMS substrates with micropatterned lines of LAM were used for the culture of PC12 cells to demonstrate neurite alignment and growth, as described below. PC12 cell growth was restricted to the LAM lines without needing to use blocking agent as commonly used with other cell types [35], [36].

### Mechanical Characterization

The six PDMS formulations were poured into 150 mm petri dishes to a thickness of 2 mm and cured for 24 hours at room temperature followed by 4 hours at 60°C. Tensile bar strips were cut using a Zing Laser Cutter (Epilog Laser, Golden, CO, USA) and uniaxial tensile testing was done on an Instron 5943 (Instron, Norwood, MA, USA). A total of 6 samples from at least 3 different preparations were analyzed per condition. Samples were stretched at a rate of 2.00 mm/min until failure. The elastic modulus of the polymers was determined from the slope of the linear regression of the stress-strain curves from 0–10%.

### Surface Roughness Analysis

PDMS coated glass coverslips were imaged using an MFP-3D-BIO atomic force microscope (AFM, Asylum Research, Santa Barbara, CA) to determine the surface roughness. All samples were imaged using AC mode in air with AC160TS cantilevers (Olympus Corporation, Center Valley, PA, USA) with a scan size of 512×512 lines over an area 20 µm×20 µm. The root mean square (RMS) roughness was calculated using the Z-sensor height signal. A total of 9 locations (3 locations on each of 3 samples) were analyzed per formulation and the average RMS roughness of each blend was statistically analyzed using a one-way ANOVA on the ranks with Tukey post hoc test (Sigma Plot, Systat Software Inc., San Jose, CA, USA).

### Water Contact angle

The relative surface energy (wettability) of each PDMS formulation was determined using water contact angle measurements. For each PDMS formulation, six PDMS coated coverslips were used as prepared and six PDMS coated coverslips were additionally coated with collagen type IV (COL4, Sigma-Aldrich CO, St. Louis, MO, USA). COL4 was adsorbed onto the PDMS by placing the coverslips PDMS side down on a 200 µL drop of 50 µg/mL COL4 for 1 hour at room temperature and then rinsed twice and stored in phosphate buffered saline (PBS) until use. Advancing contact angle analysis was performed on a Rame-Hart Contact Angle Goniometer (Rame-Hart Instrument CO, Succasunna, NJ, USA). Briefly, a 1 µL drop of ddH_2_O was placed on the surface and the average of the left and right angles was measured using DROPImage software (Rame-Hart Instrument CO, Succasunna, NJ, USA). Additional 1 µL drops were added until the contact angle no longer increased. The highest contact angle value was then determined to be the advancing contact angle for the surface. Three spots on each of the coverslips were analyzed. The six values were then averaged and a two-way ANOVA with Holm-Sidak comparison (SigmaPlot) was used to determine any statistical differences between the wettability of the different PDMS formulations with and without the COL4 coating.

### PC12 Cell Culture

PC12 cells (rat adrenal pheochromocytoma cell line, ATCC, Rockville, MD, USA) received from the supplier were designate as passage 1 and used between passage 5–10 for all subsequent experiments. The cells were maintained in RPMI-1640 Medium (ATCC) containing 10% horse serum (Sigma-Aldrich), 5% fetal bovine serum (FBS, Life Technologies, Grand Island, NY, USA), and 1% Penicillin-Streptomycin (Life Technologies) [37]. Cells were seeded at a density of 5,000 cells/cm^2^ onto either Sylgard 527 or Sylgard184 substrates micropatterned with 20 µm wide, 20 µm spaced LAM lines. The seeding media consisted of RPMI-1640 medium supplemented with 50 ng/mL nerve growth factor (Life Technologies) and 1% horse serum to induce differentiation into a neuronal phenotype. The cells were imaged on days 3, 5, 7 and 14 after seeding to determine neurite length using a Nikon TS100 phase contrast microscope equipped with a Nikon D7000 camera (Nikon Instruments Inc., Melville, NY, USA).

### C2C12 Cell Culture and Immunofluorescent Staining

Murine skeletal muscle C2C12 cells (ATCC) were cultured in growth medium consisting of Dulbecco’s modified Eagle Medium with 4500 mg/L glucose (DMEM-high glucose) supplemented with 10% FBS, 1% Penicillin-Streptomycin and 2 mM L-Glutamine (Sigma-Aldrich Co.). PDMS substrates were coated with FN by incubation with 25 µg/mL FN solution for 15 min and then washed three times with PBS. For myotube differentiation experiments, C2C12 myoblasts were seeded on the substrates at a density of 2–3×10^4^ cells/cm^2^ and grown to confluence for 24 hours. Myotube differentiation was induced by changing to differentiation medium consisting of DMEM- high glucose supplemented with 2% horse serum 1% Penicillin-Streptomycin and 2 mM L-Glutamine. After 5 days in differentiation media, cells were washed with PBS and then fixed and permeabilized in PBS containing 4% paraformaldehyde and 0.5% of Triton X-100 (Sigma-Aldrich Co.) for 15 min. After fixation, samples were incubated with in 1∶100 dilutions of monoclonal anti-myosin heavy chain (MHC) antibody (Life Technologies) and DAPI (Life Technologies) in PBS for one hour at room temperature. Samples were then washed 3 times in PBS and incubated in a 1∶100 dilution of Alexa Fluor 488 goat anti-mouse antibody (Life Technologies) for one hour at room temperature. Samples were then washed 3 times with PBS and mounted on glass slides using Prolong Gold antifade (Life Technologies). Myotubes were imaged using a Nikon AZ100 C2 laser scanning confocal microscope (Nikon Instruments, Inc.).

### Quantitative Image Analysis

The PC12 and C2C12 cells were imaged and then analyzed to quantitatively assess cell response to the different PDMS formulations. For the PC12 cells, the neurite length as a function of time was used to understand relative growth rates on Sylgard 527 versus Sylgard 184. Phase contrast images of isolated neurites from PC12 cells were collected on days 3, 5, 7, and 14 after seeding. The neurite lengths were calculated using the NeuronJ plugin for ImageJ (U. S. National Institutes of Health, Bethesda, Maryland, USA) [38], which facilitated the accurate tracing of the neurites. The average neurite length on Sylgard 527 and Sylgard 184 at each time point was compared using a Mann-Whitney Rank Sum Test (SigmaPlot). For the C2C12 cells, confocal images were analyzed to quantify the average length of MHC-positive myotubes as a function of the PDMS formulation (substrate elastic modulus). In addition, images were analyzed for the number of myotube clusters per unit area as a metric of differential cell response to the softer PDMS formulations. The myotube lengths were quantified using the segmented line tool in ImageJ. The cell density was calculated by counting the number of nuclei in each image using the particle counter tool in ImageJ and dividing by the area of the image. The myotube clusters were defined as groups of overlapping myotubes that and were quantified using the multi-point selection tool in ImageJ. The average myotube length and number of myotube clusters per unit area on the different PDMS formulations were compared using a one-way ANOVA on ranks with Dunn’s pairwise comparison (SigmaPlot).

## Results

### Mechanical Properties of Polydimethylsiloxane Formulations

**Figure 1:**
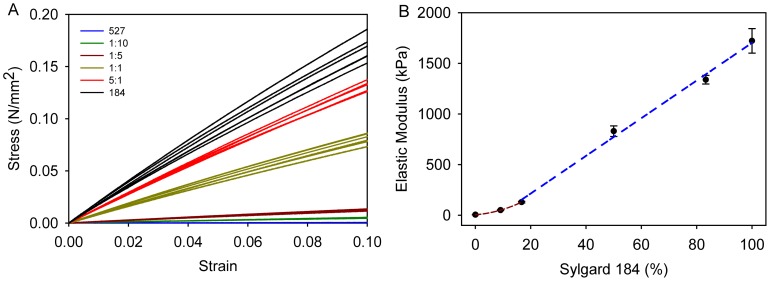
PDMS formulations span a wide range of mechanical properties from soft gels to stiff elastomers. (A) Stress strain curves for the six different PDMS formulations show that the curves for each type (n = 6) are clustered and separated from the curves of the other formulations. Over a 10% strain all formulations are linearly elastic. (B) Elastic modulus of the six different PDMS formulations as a function of weight percent Sylgard 184. The elastic modulus of each formulation is significantly different from the other PDMS formulations (One-way ANOVA, p<0.05). The curves predict that PDMS formulations can be fabricated with elastic moduli anywhere in the range from 5 kPa to 1.72 MPa by fine tuning the percentage of Sylgard 184 mixed in with the Sylgard 527. We have separated the data into two regimes, a non-linear regime for low percentages of Sylgard 184 (red curve) and a linear regime for larger percentages of Sylgard 184 (blue curve). The equation for the red curve is y = 0.3236x2+2.0606x +5 (R^2^ = 1). The equation for the blue curve is y = 18.591x–156.87 (R^2^ = 0.995). Data represented as mean ± standard deviation.

PDMS substrates were engineered by blending Sylgard 527 and Sylgard184 to tune the mechanical properties over a three order-of-magnitude range. Representative stress-strain curves (Fig. 1A) demonstrate the capability to engineer PDMS with consistent properties under uniaxial tensile loading. The curves for each formulation are linear under the range of strain investigated and are distinct, indicating that each PDMS has a different elastic modulus. The elastic modulus was determined by the slope of these curves throughout this linear regime from 0–10% strain. As expected, increasing the mass ratio of Sylgard 184 relative to Sylgard 527 increased the elastic modulus from 5.05±0.37 kPa to 1.72±0.12 MPa (Fig. 1B). The six PDMS formulations could be adjusted from soft gels to stiffer elastomers or in between by simply mixing two commercially available PDMS types, covering nearly the entire range of elastic moduli reported for soft tissues. The data for elastic modulus versus mass percent of Sylgard 184 (Fig. 1B) can be interpreted to fall into two regimes. From 0–20% Syglard 184, the data is best fit by a 2^nd^ order polynomial where the addition of small amounts of Sylgard 184 to the Sylgard 527 causes a nonlinear increase. From 20–100% Sylgard 184 the data is best fit by a linear regression where the addition of Sylgard 184 to the Sylgard 527 causes a linear increase. These two curves enable determination of the approximate mass ratio of Sylgard 184 and Sylard 527 required to create substrates with any elastic modulus within the tunable range. To simplify our terminology, we will subsequently refer to the PDMS formulations by the mean elastic modulus measured for each mass ratio; specifically Sylgard 527 = 5 kPa, 10∶1 = 50 kPa, 5∶1 = 130 kPa, 1∶1 = 830 kPa, 1∶5 = 1.34 MPa and Sylgard 184 = 1.72 MPa.

### Surface Roughness

**Figure 2:**
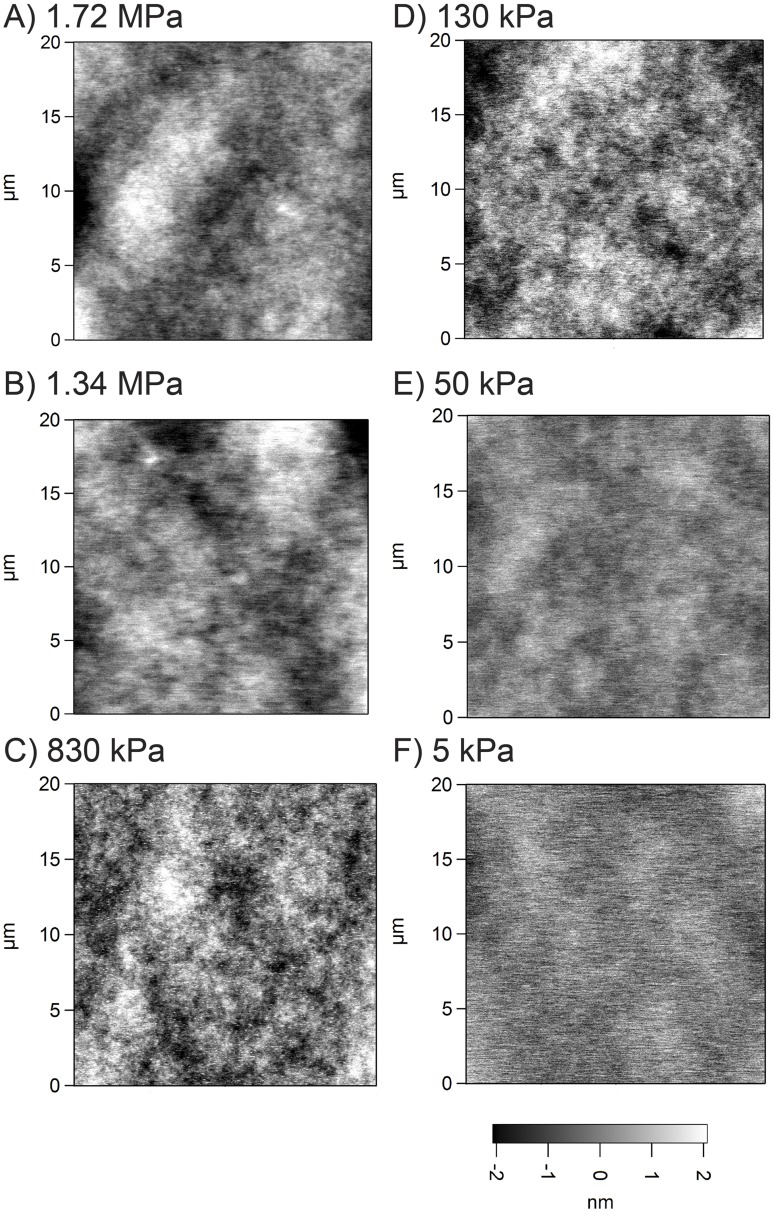
Representative AFM scans of the surface topography for the different PDMS formulations. These images show that all PDMS formulations have similar morphological appearance and total variation in height of ∼4 nm over a 20 µm scan area. The different scans are for (A) 1.72 MPa, (B) 1.34 MPa, (C) 830 kPa, (D) 130 kPa, (E) 50 kPa and (F) 5 kPa elastic modulus PDMS formulations.

**Figure 3:**
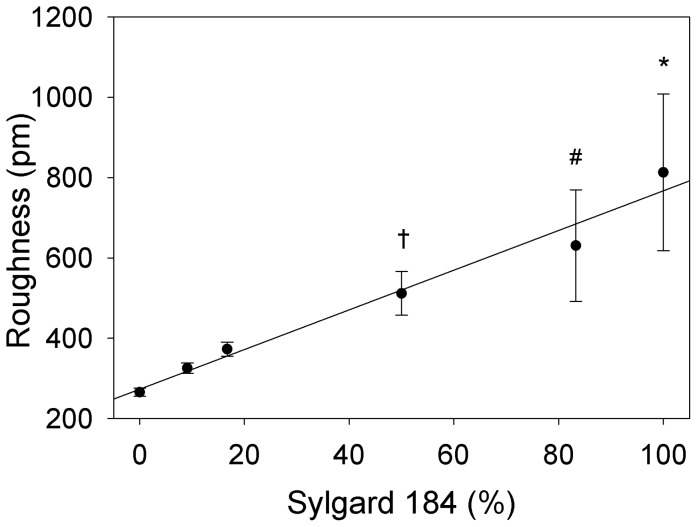
RMS roughness of the six PDMS formulations as a function of weight percent Sylgard 184. As the percentage of Sylgard 184 increases, the RMS roughness also increases, ranging from approximately 200 to 800 pm. While there are significant difference in roughness between formulations, all have an RMS roughness of <1 nm, smaller than what cells can typically differentiate. Thus, we consider all the PDMS formulations to have equivalent surface roughness in terms of what a cell can sense and respond to. The relationship between RMS roughness and weight percent Sylgard 184 is fit by a linear regression (solid line, y = 273.25 + 4.94x, R^2^ = 0.9745). Data represented as mean ± standard deviation, statistical significance determined by one-way ANOVA on the ranks with Tukey post hoc test (n = 9) where (*) was significantly different from 0, 9.09 and 16.67%, (#) was significantly different from 0 and 9.90% and (†) was significantly different from 0% Sylgard 184 formulations (p<0.05).

We evaluated the surface roughness to determine whether there was a difference between the PDMS formulations that might influence cell response. AFM was used to analyze the surface topography and generate height maps in order to calculate the RMS roughness. All the formulations had a similar appearance over a square 20 µm scan size (Fig. 2). It should be noted that for the four PDMS blends there were no indications of phase separation between the Sylgard 184 and Sylgard 527, appearing to be completely miscible in one another as expected. Further, the fumed silica nanoparticles in the Sylgard 184 did not alter the surface morphology, with all samples generally varying in height no more than 4 nm over the scan area. The RMS roughness of the PDMS increased linearly with elastic modulus (Fig. 3). Statistical analysis using one-way ANOVA on the ranks with Tukey post hoc test indicated that the PDMS with elastic modulus of 1.72 MPa had a higher RMS roughness compared to the 5, 50 and 130 kPa formulations, that 1.34 MPa PDMS had a higher RMS roughness compared to 5 and 50 kPa formulations; and that 830 kPa PDMS had a higher RMS roughness compared to 5 kPa PDMS (Fig. 3). However, the RMS roughness was <1 nm for all the PDMS formulations, which is generally considered below the detectable range of cells [39]. Note that it remains possible that the variation in RMS roughness may be an artifact of the AFM probe compressing the softer PDMS formulations more, but should still be <1 nm for all conditions. Thus, these results suggest that the surface roughness is equivalent in terms of biological affect across the entire range of elastic moduli.

### Surface Wettability

**Figure 4:**
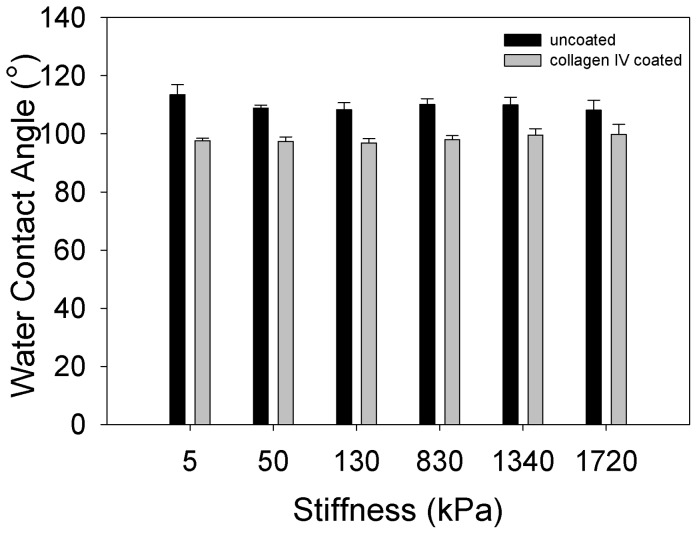
Water contact angle of uncoated and collagen IV coated PDMS formulations. The water contact angles of all uncoated PDMS formulations (black) are approximately 110°, indicating a similar surface energy and hydrophobicity. The water contact angles of all PDMS formulations decreases to approximately 100° when coated with collagen type IV (gray), indicating similar protein adsorption behavior and surface energy. All uncoated PDMS formulations were relatively hydrophobic thought to be indistinguishable to cells despite the small, but statistically significant differences in water contact angle between the 5 kPa versus the 1.72 MPa, 130 kPa and 50 kPa substrates (# indicates p<0.05). All collagen type IV coated PDMS formulations were statistically equivalent and had statistically significant decreases in water contact angle compared to the uncoated PDMS (*indicates p<0.05). Data represented as mean ± standard deviation, statistical significance based on two-way ANOVA with Holm-Sidak pairwise comparison (n = 6).

The surface energy of a substrate can affect the types and amounts of proteins that are able to adhere to the surface, affecting cell adhesion and behavior [40], [41]. We used water contact angle measurements to determine whether the surface energy was constant for the different PDMS formulations (Fig. 4). The water contact angle of the uncoated PDMS was ∼110° for all formulations, indicating a hydrophobic surface and comparable to previously reported values for Sylgard 184 and other types of PDMS [27], [42]. There were statistically significant differences in the water contact angle between some of the PDMS formulations, but these did not follow a distinct pattern and were always between 105° and 110°, a difference that is likely below what a cell can sense given that all samples are relatively hydrophobic in nature compared to a cell membrane. Coating the PDMS with COL4 increased the hydrophilicity and decreased the water contact angle to ∼100° for all the formulations. An important note is that after protein coating with COL4 there were no statistically significant differences in the water contact angle between any of the conditions. Thus, even though small differences in contact angle were present before protein coating, after protein coating all the surfaces were comparable. Similar to the surface roughness, these results suggest that the surface energy after ECM protein coating is constant across the entire range of substrate elastic moduli.

### Microcontact Printing of Extracellular Matrix Proteins onto the Polydimethylsiloxane Formulations

**Figure 5:**
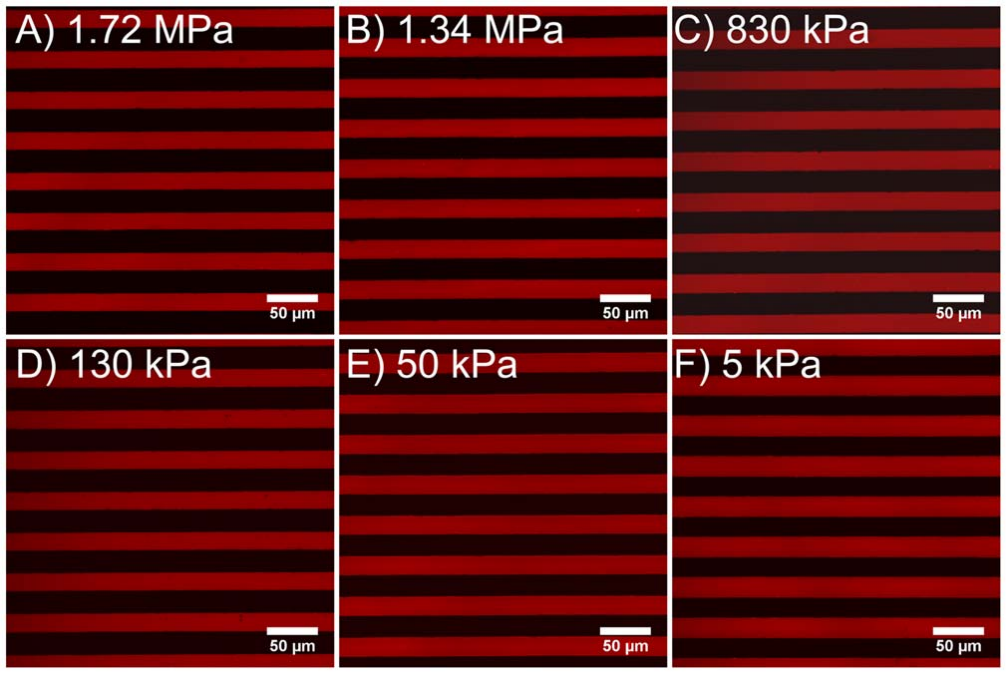
Representative examples of fluorescently labeled fibronectin micropatterned on the different PDMS formulations. The protein pattern is transferred with high fidelity on all the PDMS formulations indicating the substrates exhibit similar protein adsorption from the PDMS stamps (made from Sylgard 184) used for microcontact printing. The different images are for (A) 1.72 MPa, (B) 1.34 MPa, (C) 830 kPa, (D) 130 kPa, (E) 50 kPa and (F) 5 kPa elastic modulus PDMS formulations. Scale bars are 50 µm.

**Figure 6:**
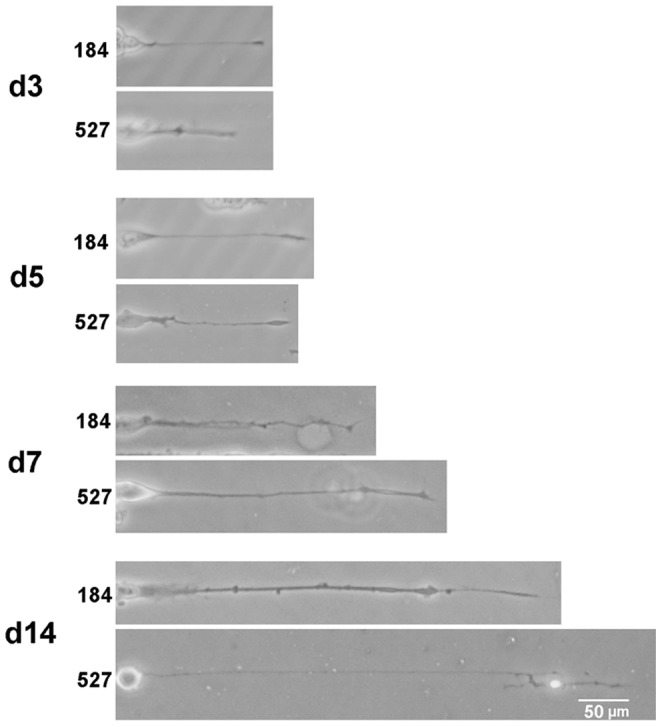
Representative phase contrast images show single neurites extending from the cell body of PC12 cells. The PC12 cells were differentiated into neuron-like cells and cultured on 5 kPa and 1.72 MPa PDMS (Sylgard 527 and Sylgard 184, respectively). Laminin was micropatterned as 20 µm wide, 20 µm spaced lines to direct the linear extension of neurites, which were imaged at 3, 5 7 and 14 days. The neuron length increased with culture time and was qualitatively similar between the two PDMS types. Scale bar is 50 µm.

PDMS substrates are often micropatterned with ECM proteins in order to control the way cells adhere and interact. Soft substrates such as gels with an elastic modulus of <100 kPa have been difficult to pattern with techniques such as microcontact printing, instead requiring additional fabrication steps [43], [44]. Here we show that microcontact printing was able to transfer FN onto each of the PDMS formulations with high fidelity (Fig. 5). The 20 µm wide, 20 µm spaced FN lines were well transferred with no apparent difference in the uniformity of protein coating. Additionally, we created the same line micropattern using LAM instead of FN on Sylgard 184 and Sylgard 527 (Fig. 6). Here we did not stain for the ECM protein, but rather evaluated bioactivity of the LAM via directed neurite extension of PC12 cells. The LAM patterns maintained anisotropic neurite extension for 14 days in culture demonstrating that the PDMS surfaces are able to maintain attachment of the LAM over this time and in the presence of FBS. It should be noted that the PDMS surfaces maintained the patterned ECM proteins over prolonged culture periods even though the ECM proteins were not covalently linked to the PDMS.

### Rate of Neurite Extension on Hard and Soft Polydimethylsiloxane

**Figure 7:**
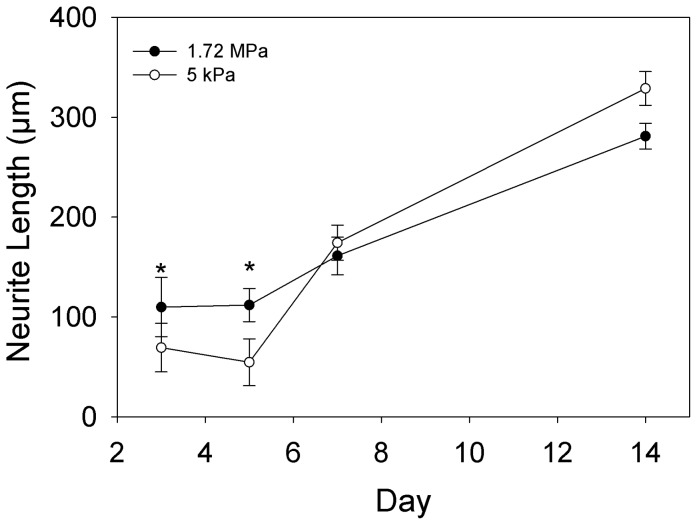
Quantification of neurite length for PC12 cells cultured on two different PDMS formulations. PC12 cells were cultured on 1.72 MPa (•, black circles) and 5 kPa (○, white circles) PDMS and evaluated at days 3, 5, 7 and 14. At days 3 and 5, neurite length on 1.72 MPa PDMS was significantly greater compared to neurite length on 5 kPa PDMS. On days 7 and 14 the neurite length was equivalent on both PDMS types. This suggests that PC12 neurites initially grow faster on stiffer PDMS substrates (up to 5 days), but by 7 days the growth rate has slowed on the stiffer PDMS and accelerated on the softer PDMS such that neurite lengths are equivalent. Data represented as mean ± standard error of the mean. Statistical significance at each time point determined by a Mann-Whitney Rank Sum Test, *indicates p≤0.001.

The PC12 cell line is widely used as model system because these cells are able to differentiate into neuronal-like cells that extend neurites. Previous reports have indicated that brain tissue has an elastic modulus of 0.1–1 kPa [1], [45]. Thus, we used PC12 cells differentiated into neurons to determine if the rate of neurite extension was sensitive to the underlying substrate mechanics. Sylgard 527 (*E* = 5 kPa) served as our brain-like stiffness and Sylgard 184 (*E* = 1.72 MPa) served as our much stiffer material for comparison. We did not investigate intermediate elastic moduli because preliminary studies (data not shown) indicated minimal differences between many of the intermediate formulations. Because neurons will extend neurites in complex, isotropic orientations, we chose to micropattern the PDMS surfaces with 20 µm wide lines of LAM in order to direct uniaxial neurite extension and facilitate measurement of neurite length. Success of the LAM patterning was demonstrated by the linear neurite growth (Fig. 6). The PC12 cells were differentiated into neurons and phase-contrast images were recorded on days 3, 5, 7 and 14 (Fig. 6). Quantification using ImageJ revealed a statistically significant increase in neurite length on Sylgard 527 versus Sylgard 184 at days 3 and 5, but by days 7 and 14 neurite length was statistically equivalent on both substrates (Fig. 7). This suggests that at early time points up to 5 days, neurites extend more rapidly on softer substrates with an elastic modulus more similar to brain tissue, however at longer time points the neurites on the stiffer PDMS appear to catch up such that neurite lengths are comparable by 7 and 14 days. The implication is that cells may have a transient response to substrate mechanics that affects growth kinetics, but that this difference may disappear over time as neurites reach a maximal length in culture.

### Myogenesis on Variable Stiffness PDMS Substrates

**Figure 8:**
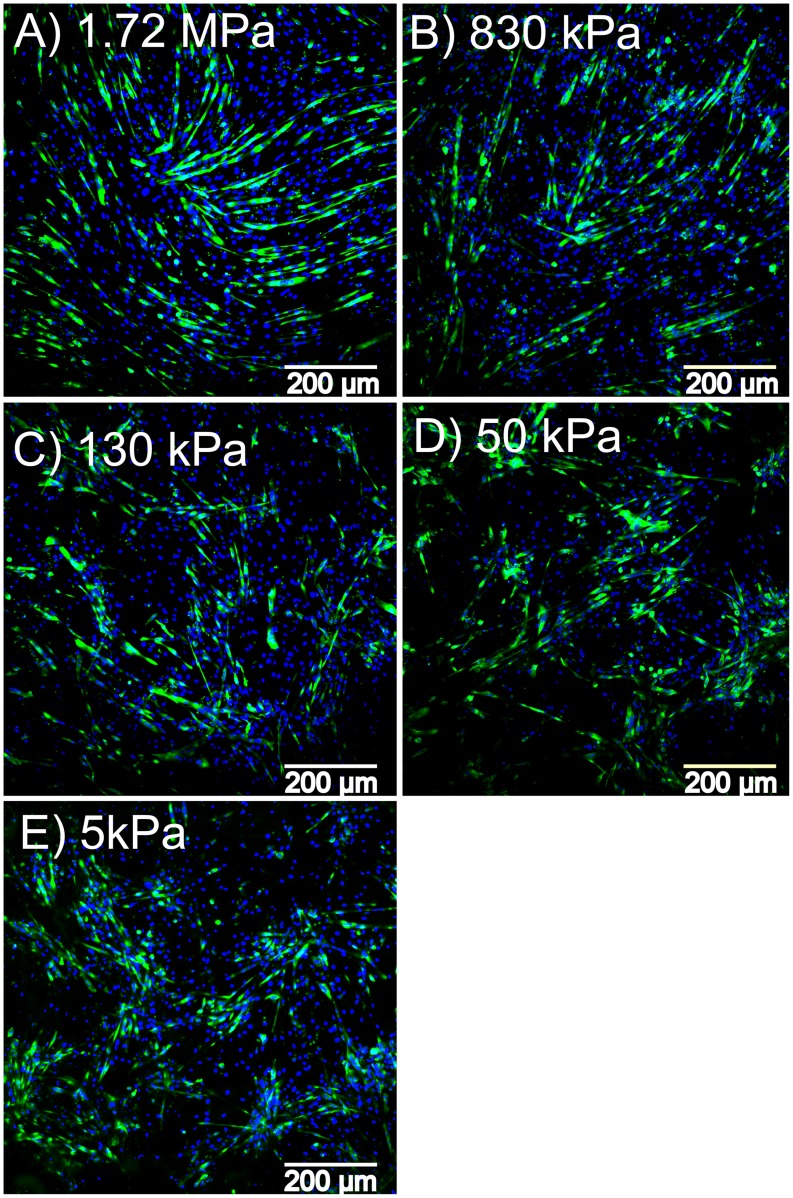
Representative fluorescent images of C2C12 cells differentiated into myotubes on different PDMS formulations. C2C12 cells cultured and differentiated on PDMS (A) 1.72MPa, (B) 830 kPa, (C) 130 kPa, (D) 50 kPa and (E) 5 kPa formulations. All cells were stained for the nucleus with DAPI (blue) and differentiated myotubes were stained for myosin heavy chain (green). Cells cultured on the stiffer PDMS substrates (A–C) formed longer myotubes, whereas cells cultured on the softer substrates (D and E) formed shorter myotubes and tended to organize into cell clusters. Scale bars are 200 µm.

**Figure 9:**
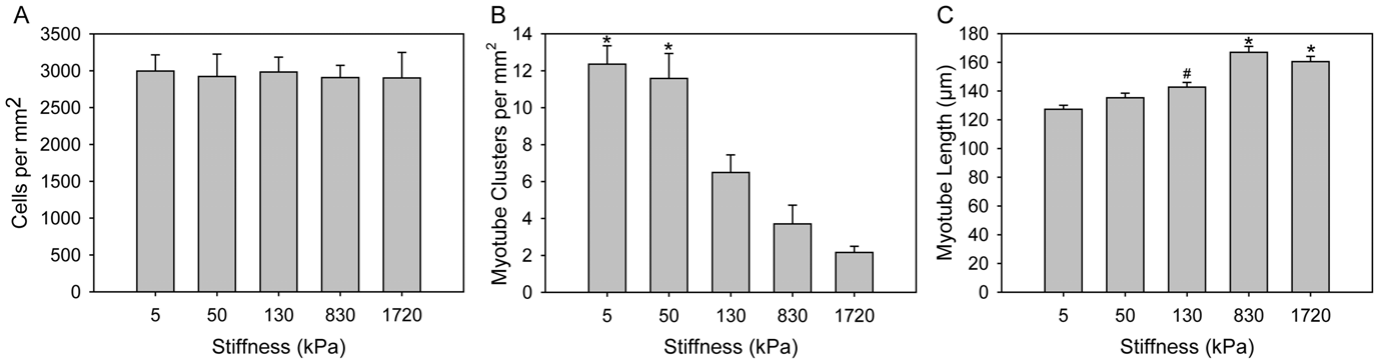
Quantification of cell density, myotube length and myotube clustering performed as a function of the PDMS elastic modulus. (A) Average cell density of the different PDMS formulations shows no difference as a function of substrate elastic modulus. (B) Average number of myotube clusters per mm^2^ on the different PDMS formulations (n = 9). The cells cultured on the 5 and 50 kPa substrates formed significantly more myotube clusters compared to the other substrates (*indicates p<0.001). (C) Average length of myosin heavy chain positive myotubes on the different PDMS formulations (5 kPa, n = 706; 50 kPa, n = 739; 130 kPa, n = 662; 830 kPa, n = 769; 1.72 MPa, n = 760). Cells cultured on the stiffer 1.72 MPa and 830 kPa substrates formed significantly longer myotubes compared to those formed on the softer 130, 50 and 5 kPa substrates (*indicates p<0.001). Cells cultured on the 130 kPa substrate also formed longer myotubes compared to those formed on the 5 kPa substrate (# indicates p<0.001). Data represented as mean ± standard error of the mean, statistical analysis by Kruskal Wallis ANOVA on the ranks with p<0.05 Dunn’s method for pairwise comparison.

The C2C12 cell line is widely used to study myotube formation and has also been shown to differentiate into other cell types such as osteoblasts and adipocytes based on soluble cues and substrate mechanics [46]–[48]. Based on this, we studied the differentiation of the C2C12 myoblasts into myotubes on five of the PDMS formulations with elastic moduli of 1.72 MPa, 830, 130, 50 and 5 kPa. The surfaces were coated with FN to increase cell adhesion. Cells were cultured to confluence in growth media, differentiated for 5 days and then fixed and stained with MHC to visualize the myotubes and DAPI to identify cell nuclei. Results show that all PDMS formulations supported C2C12 adhesion, proliferation and differentiation into multinucleated myotubes (Fig. 8). While myotubes formed on all the surfaces, there were differences in myotube length and multicellular organization as a function of substrate elastic modulus. Myotubes on the stiffer PDMS (Fig. 8A and 8B) were locally organized in parallel with each other, similar to that observed for these cell cultured on tissue culture polystyrene [49]. However, myotubes on the softer PDMS (Fig. 8C to 8E) formed myotube clusters where parts of many myotubes overlapped with each other and local alignment between myotubes was not apparent. Sylgard 527 has been used infrequently in the literature for cell culture [50], thus we wanted to verify that Sylgard 527 and the blends did not have increased cytotoxicity relative to the standard Sylgard 184. We quantified the number of C2C12 cell nuclei per unit area (cell density) on the different PDMS surfaces and demonstrated that there is no significant difference between formulations (Fig. 9A). The ability of Sylgard 527 to support equivalent cell density to Sylgard 184 after 6 days in culture strongly suggests equivalent biocompatibility between these two types of PDMS. Quantifying the number of myotube clusters that formed as a function of elastic modulus (Fig. 9B) revealed that this behavior increased for the softer materials, and appeared to reach a maximum for elastic moduli in the range of 5 to 50 kPa. This type of clustering behavior generally occurs when cells prefer adhesion to each other rather than to the substrate. In the case of these cells it suggests that substrate elastic modulus can regulate the preference between cell-cell adhesion and cell-substrate adhesion, but determining what the actual underlying mechanism is requires additional future studies. The substrate stiffness also impacted the average myotube length, with myotubes on stiffer PDMS ∼20% greater in length relative to softer PDMS (Fig. 9C). It is probable that the decreased myotube length and clustering behavior on the soft PDMS are coupled responses to substrate mechanics that have their basis in altered cytoskeletal structure, as previous studies have shown this type of mechanosensitivity in the cytoskeleton of C2C12 myotubes [51].

## Discussion

PA gels have been the *de facto* standard for studying cell response to substrates with elastic modulus in the range of 1 to 100 kPa [1], [2], [6], [11]–[15]. This range is comparable to the elastic modulus of many soft tissue types, but there are also many soft tissues that have much higher reported elastic moduli. Studies with stiffer materials have demonstrated that cells are also sensitive to differences in substrate elastic modulus in this higher range from 100 kPa to 1 MPa [23]–[26]. Unfortunately, it has been difficult to study cell response continuously across the entire elastic modulus range of soft tissues from 1 kPa to 1 MPa because it required using different materials with different chemical and physical properties. Here we have demonstrated that PDMS formulations formed by blending together Sylgard 527 and Sylgard 184 are able to cover this entire range and that the elastic modulus can be tuned independently of other material properties such as surface chemistry, energy and roughness.

While Sylgard 184 has been used in a large number of cell culture studies, Sylgard 527 has been used infrequently [50] and there is a potential concern that it may not be biocompatible. To address this, we used C2C12 cells and demonstrated that after 6 days in differentiation media the cell density on Sylgard 527, Sylgard 184 and blends of the two were all statistically equivalent (Fig. 9A). This strongly suggests that there is no increased cytotoxicity associated with Sylgard 527. This makes sense based on the polymer chemistry, because Sylgard 527 and Sylgard 184 are primarily the same material consisting of dimethylvinyl-terminated dimethyl siloxane and dimethyl, methylhydrogen siloxane, with the main difference being that Sylgard 184 has a silica nanoparticle filler [52]. While the exact composition of Sylgard 527 and Sylgard 184 are proprietary, the materials safety data sheets (MSDS) for each PDMS provides detail on the chemical components and those that are potentially cytotoxic [53]–[55]. For Sylgard 527 parts A and B, the MSDS states that they contain 85 to 100 wt% dimethylvinyl-terminated dimethyl siloxane and 1 to 5 wt% dimethyl, methylhydrogen siloxane. In contrast, the widely used Sylgard 184 contains more potentially cytotoxic chemicals. For Sylgard 184 Base resin, the MSDS states that it contains 0.5 wt% xylene, 0.2 wt% ethylbenzene, >60 wt% dimethylvinyl-terminated dimethyl siloxane, 30 to 60 wt% dimethylvinylated and trimethylated silica and 1 to 5 wt% tetra(trimethylsiloxy) silane. For Sylgard 184 Curing Agent, the MSDS states that it contains 0.19 wt% xylene, <0.1 wt% ethylbenzene, 55 to 75 wt% dimethyl, methylhydrogen siloxane, 15 to 35 wt% dimethylvinyl-terminated dimethyl siloxane, 10 to 30 wt% dimethylvinylated and trimethylated silica and 1 to 5 wt% tetramethyl tetravinyl cyclotetrasiloxane. These chemical compositions demonstrate that Sylgard 184 contains the two main siloxanes in Sylgard 527 plus additional chemicals and fillers including the solvent xylene, the carcinogen ethylbenzene and silica nanoparticles. While the MSDS does not provide complete information on chemical composition, it is clear that Sylgard 527 and the blends with Sylgard 184 all have the same basic siloxane chemistry and that there are no chemicals in Sylgard 527 that would increase its cytotoxicity relative to the widely used Sylgard 184.

The blends we have developed offer distinct advantages over previously reported methods to tune the elastic modulus of PDMS. The most common technique to decrease the elastic modulus of Sylgard 184 has been to decrease the ratio of curing agent to base resin from the manufacturer’s recommendation of 1∶10 to as low as 1∶70 [32], [56]–[62]. This decreases the crosslink density, but is not ideal for a number of reasons. The first limitation of this approach is that the recommended 1∶10 ratio is designed to optimize the stoichiometry of the crosslinking reaction. Reducing the relative amount of crosslinker increases the amount of free, non-crosslinked PDMS chains in the polymer matrix that are able to diffuse out of the bulk material. Because PDMS linear chains have lower surface energy than crosslinked PDMS, there is a driving force for the non-crosslinked PDMS to segregate to the surface of the bulk PDMS [63]. This results in the formation of an oil-like layer of oligomeric PDMS on the surface [64], [65] that can potentially disrupt cell adhesion and other processes [66]. The long-term consequence of cellular uptake of this oligomeric PDMS chains remains an unresolved area of concern. The second problem is that Sylgard 184 is filled with fumed silica, glassy nanoparticles that add significant stiffness to the polymer and accounting for 30 to 60 wt% of the cured polymer [52], [54], [55]. These nanoparticles are in both the base resin and curing agent, so reducing the curing agent content does not remove these nanoparticles. As a result, the decreased elastic modulus of the 1∶70 Sylgard 184 requires an extremely low crosslink density and a large number of free chains to compensate for the stiff nanoparticles, exacerbating the problem of free oligomer chains highlighted above. In contrast, Sylgard 527 does not contain any fumed silica filler or other reinforcements. Mixing the A and B components in the recommended 1∶1 ratio produces a PDMS with polymer chains that are crossslinked into the network, yet have a very low elastic modulus (∼5 kPa). Mixing increasing amounts of Sylgard 184 into Sylgard 527 achieves stiffer formulations that maintain the stoichiometry of the individual PDMS types while providing good control over the mechanical properties.

Researchers have also explored other approaches to control the crosslink density of PDMS. For example, trimethyl terminated PDMS oils have been incorporated into the PDMS while it is cured, which are unable to covalently crosslink via hydrosilation curing and thus decrease the crosslink density of the elastomer network [67]. Using this strategy, the elastic modulus of Silastic T2, another silica-filled PDMS, was reduced to as low as ∼800 kPa and the fact that the PDMS oils leached out was used to enhance the fouling release characteristics of the PDMS. Another approach used to modify crosslink density has been controlling the temperature and time at which Sylgard 184 has been cured. For example, varying the baking time from 15 minutes to 3 days and the curing agent from 3% to 10% enables tuning the elastic modulus of Sylgard 184 from 50 kPa to 4,000 kPa [68]. While this is a large range, the soft PDMS continues to cure at room temperature and thus the mechanical properties are not stable with time. This makes it problematic for cell culture studies where the PDMS will be placed in a 37°C incubator for prolonged periods of time and the elastic modulus will increase. With the goal of spatially patterning the mechanical properties, Sun *et al* have developed an approach that uses benzophenone added to the Sylgard 184 as a photo initiator to reduce the crosslink density when exposed to UV light [69]. This can produce Sylgard 184 with an elastic modulus as low as 27 kPa when formulated with a base to curing agent ration of 30∶1 and short curing times of 20 minutes at 110°C. Uniquely, the UV exposure also stabilizes the reduced crosslink density and thus the photo-sensitive PDMS mechanical properties do not change over time. While this approach cannot achieve an elastic modulus as low as the 5 kPa we report with our blend systems, it does suggest that incorporation of benzophenone into our system may enable spatially patterning of PDMS stiffness in this range, and perhaps decreasing the elastic modulus even further.

An additional advantage of our approach is the consistent mechanical properties and surface properties we achieved from batch to batch. Contrast this to the substantial variability in the literature for the reported elastic modulus of Sylgard 184 with reduced curing agent ratios (Fig. S1). Summarizing the results from ten different studies shows that the standard Sylgard 184 formulation with 1∶10 curing agent to base ratio has reported elastic moduli ranging from 1 to 2.5 MPa [32], [56], [57], [60]–[62], [66], [70], [71], though most are similar to our value of *E* ∼1.7 MPa. Reducing the curing agent ratio produces varying results that makes it difficult to choose the optimum formulation to achieve a PDMS with a specific elastic modulus. For example, the ratio of 1∶50 has reported elastic moduli of 8, 12, 30 and 48 kPa, a 600% difference between the lowest and highest values [32], [57], [61], [62]. Similarly, to tune the elastic modulus to ∼10 kPa reveals conflicting reports on whether 1∶50, 1∶55 or 1∶67 is the appropriate curing agent to base ratio [32], [57], [58], [62]. It remains unclear what underlies this reported variability, but it likely includes multiple factors such as differences in curing time and temperature and accurate measuring and mixing of small quantities of curing agent. Our experimental protocol avoids some of these potential issues by never combining two components in less than a 1∶10 ratio and using a conforming mixer to ensure optimal mixing of our PDMS.

The C2C12 and PC12 cell lines both demonstrated mechanosensitive cellular responses to variation of the substrate elastic modulus, verifying the effectiveness of our tunable PDMS system. The C2C12 cells differentiated into myotubes on all of the PDMS surfaces, with a maximum myotube length on the 830 kPa substrate and a minimum on the 5 kPa substrate (Fig. 9C). While a number of studies have looked at various C2C12 differentiation metrics as a function of substrate mechanics, none have examined an elastic modulus range as large as we have. For example, Engler *et al* micropatterned lines of myotubes on PA gels and showed enhanced sarcomere formation on an elastic modulus of 11 kPa compared to gels only ∼7 kPa softer or stiffer [51]. What complicates comparison of our results to these micropatterned myotubes is that C2C12 cells cultured as 2-D sheets form myotubes on top of a layer of non-differentiated cells, which are the ones that are actually adhered to the substrate, which Engler *et al* has shown may be obscure the effect from the underlying substrate stiffness [51]. However, it is evident from our studies that myotube length (Fig. 9B) and myotube clustering (Fig. 9C) were sensitive to order-of-magnitude changes in substrate elastic modulus. This is in general agreement with Xu *et al*, who used variable stiffness silk-based materials to show enhanced C2C12 proliferation and Myo1d expression on substrates with an elastic modulus of 20 MPa compared to 25 MPa and 5 MPa substrates [72]. Using muscle progenitor cells rather than C2C12 cells, Boonen *et al* compared 3 kPa and 21 kPa PA gels and glass coverslips with various ECM coatings and generally found that myotube differentiation was best on the glass coverslip (elastic modulus >10 GPa) [73], [74]. Considering all these results, what is clear is that differentiation of myoblasts into myotubes is sensitive to substrate mechanics, but it is not a simple relationship and that cell type, micropatterning, materials type and ECM coating all play a role. For comparison, the PC12 cells grew longer neurites on the 5 kPa substrate at 3 and 5 day time points, but by 7 days neurites on the 1.72 MPa substrate had reached the same length and continued to increase in length out to 14 days. What this shows is that PC12 cells initially extend neurites farther on the softer substrate, but that this response to substrate mechanics is time dependent. Cheng *et al* showed similar results where PC12 cells differentiated for 6 days extended longer neurites on soft gelatin and gelatin-chitosan composite substrates than on stiffer chitosan substrates [75]. Using very soft PA gels ranging from 7 Pa to 19 kPa, Leach *et al* showed an increase in neurite length for the stiffer substrates [76], but even the stiffest PA gels were similar to the 5 kPa PDMS we used in our studies. In total, what our results show is that PC12 and C2C12 cells are responsive to the tunable PDMS over the three order-of-magnitude range we have to work with. Our results are in general agreement with previous studies, but it should be noted that every one of these studies uses a unique combination of materials, ECM protein coatings, range of substrate elastic modulus and use or absence of micropatterning. Looking forward, this is specifically where our tunable PDMS system is advantageous because it provides a simple, robust platform for specifically varying elastic modulus without altering other surface properties, and the same micropatterning techniques can be used across the entire stiffness range.

### Conclusions

We have demonstrated that PDMS formulations based on the blending of commercially available Sylgard 527 and Sylgard 184 are able to create biomaterials with tunable elastic modulus over three orders-of-magnitude. This enables independent control of mechanics with minimal effect on surface roughness, surface energy, the ability to absorb ECM proteins from solution or the ability to be micropatterned with ECM proteins. This is an improvement over what have been previously reported using PA gels and PDMS elastomers. Our cell studies demonstrate that all formulations support adhesion, growth and differentiation and that cell behaviors such as neurite extension and length of differentiated myotubes are sensitive to the differences in substrate elastic modulus. Our PDMS formulation are widely applicable to the study of cell response to variable substrate mechanics and have the advantage of being reproducible and simple to fabricate from commercially available, low cost PDMS while covering the largest range of physiologically-relevant elastic modulus currently reported in the literature.

## Supporting Information

Figure S1 **Summary plot of published values for the elastic modulus of Sylgard 184.** In these examples the manufacturer’s recommend ratio of curing agent to base has been reduced from 1∶10 down to as low as 1∶70 to vary the elastic modulus of the Sylgard 184. Variability of greater than 250% in the measured elastic modulus is observed at the commonly used ratios of 1∶10, 1∶20, 1∶30 and 1∶50. Values are included from Cheng *et al* 2009 (•), Brown *et al* 2005 (○), Gray *et al* 2003 (▾), Ochsner *et al* 2007 (Δ), Bartalena *et al* 2011 (▪), Ahmed *et al* 2011 (□), Liao *et al* 2010 (♦), Wang *et al* 2010 (◊), Evans *et al* (▴) and Tzvetkova-Chevolleau *et al* () [32], [56]–[62], [70], [71]. The inset shows the elastic modulus for curing agent to base ratios of 1∶50 to 1∶100, note the variability of greater than 500% at the commonly used value of 1∶50 (∼2 weight percent curing agent).
(TIF)
